# Structural and antitumoral characteristic dataset of the chitosan based magnetic nanocomposite

**DOI:** 10.1016/j.dib.2019.104583

**Published:** 2019-09-28

**Authors:** Hamed Tashakkorian, Vahid Hasantabar, Monire Golpour

**Affiliations:** a Cellular and Molecular Biology Research Center (CMBRC), Health Research Institute, Babol University of Medical Sciences, Babol, Iran; b Department of Pharmacology, School of Medicine, Babol University of Medical Sciences, Babol, Iran; c University of Mazandaran, Faculty of Chemistry, Department of Organic-Polymer Chemistry, Babolsar, 47416, Iran; d Molecular and Cell Biology Research Center, Student Research Committee, Faculty of Medicine, Mazandaran University of Medical Sciences, Sari, Iran

**Keywords:** Magnetic nanobiocomposite, Click chemistry, Antitumoral assay, Structural data, Cell lines

## Abstract

The evaluation on the characteristic dataset and figures presented here, are related to our latest research data entitled “Fabrication of chitosan based magnetic nanocomposite by click reaction strategy; evaluation of nanometric and Cytotoxic characteristics” [1]. FTIR, Vibrating Sample Magnetometer (VSM) measurements, Xray diffraction (XRD) information and the resulted figures for structural confirmation of the prepared chitosan based nanocomposite are presented in this article. The morphological changes of the Fibroblast, Saos, MCF7 and Hela cell lines after treatment with the mention compound were displayed. The additional adsorption data for the synthesized nanobiocomposite were also demonstrated with graphs.

**Table:** Specifications Table

Subject area	*Chemistry, Biology*
More specific subject area	*Preparation of chitosan based nanobiocomposite*
Type of data	*raw data, graph, figure*
How data was acquired	*The outcomes were provided by IR, VSM, XRD and MTT assay. Also some descriptions about the composite preparation and images of the morphology of cell lines were presented.*
Data format	*Raw, analyzed*
Experimental factors	*FTIR of the prepared samples, VSM and XRD of the composite, also the images of cell lines were apprised.*
Experimental features	*The nanocomposite was prepared and characterized using FTIR and imaged by SEM and TEM technique. Then thermophysical experiments were performed using DSC and TGA protocols. Biological characteristics were evaluated with MTT assay and the morphological effects were imaged by microscopic technique.*
Data source location	*Babol university of medical sciences, Mazandaran, Iran*
Data accessibility	*Available in this article*
Related research article	*Fabrication of chitosan based magnetic nanocomposite by click reaction strategy; evaluation of nanometric and Cytotoxic characteristics* [1]

**Table:** 

**Value of the Data** • *This data presents the structural and physical characteristics of the synthesized biocomposite, from which researchers who are interested in preparation of novel chitosan based nanocomposite especially in medical field can take advantage of it.* • *The isotherm linear absolute and isotherm pressure composition plots data which is introduced as tables and figure can gain the attention of the chemical and environmental engineers for production of the new class of bioadsorbents.* • *The biocharacteristics of the prepared composite toward Fibroblast, MCF7, Hela, and Saos cell lines were investigated and the data can encourage researches towards assessments against other cancer cell typs.*

## 1 Data

**Fig. 1:**
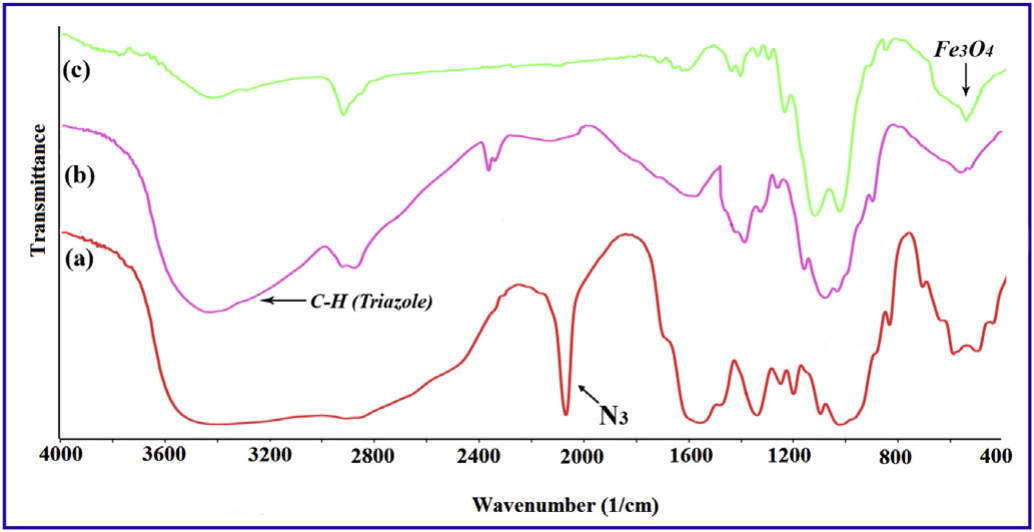
FT-IR spectra of a) azidated chitosan, b) chitosan-silane composite, c) chitosan-silane clicked @ Fe_3_O_4_.

**Fig. 2:**
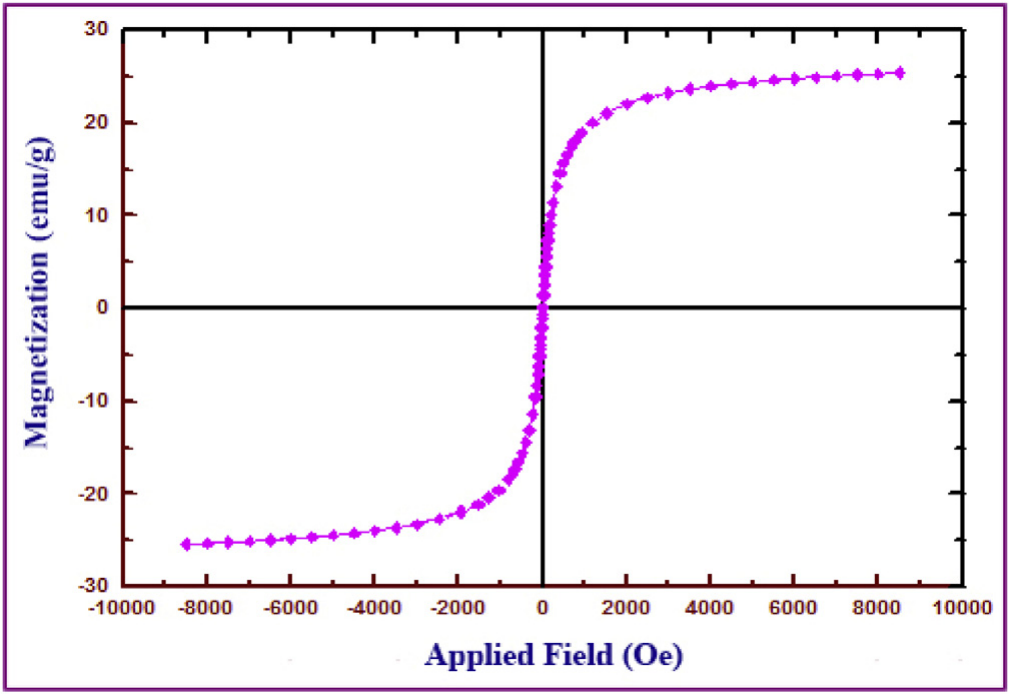
Magnetization curves of Fe_3_O_4_@functionalized chitosan nanobiocomposite.

**Fig. 3:**
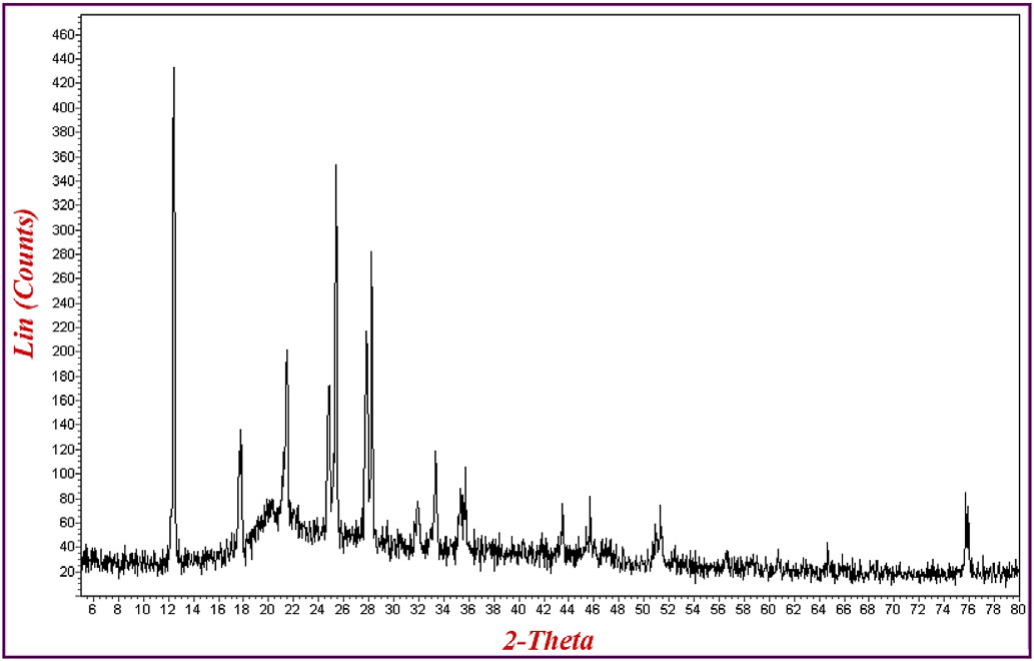
XRD patterns of the synthesized SiO_2_/functionalized chitosan composite.

**Fig. 4:**
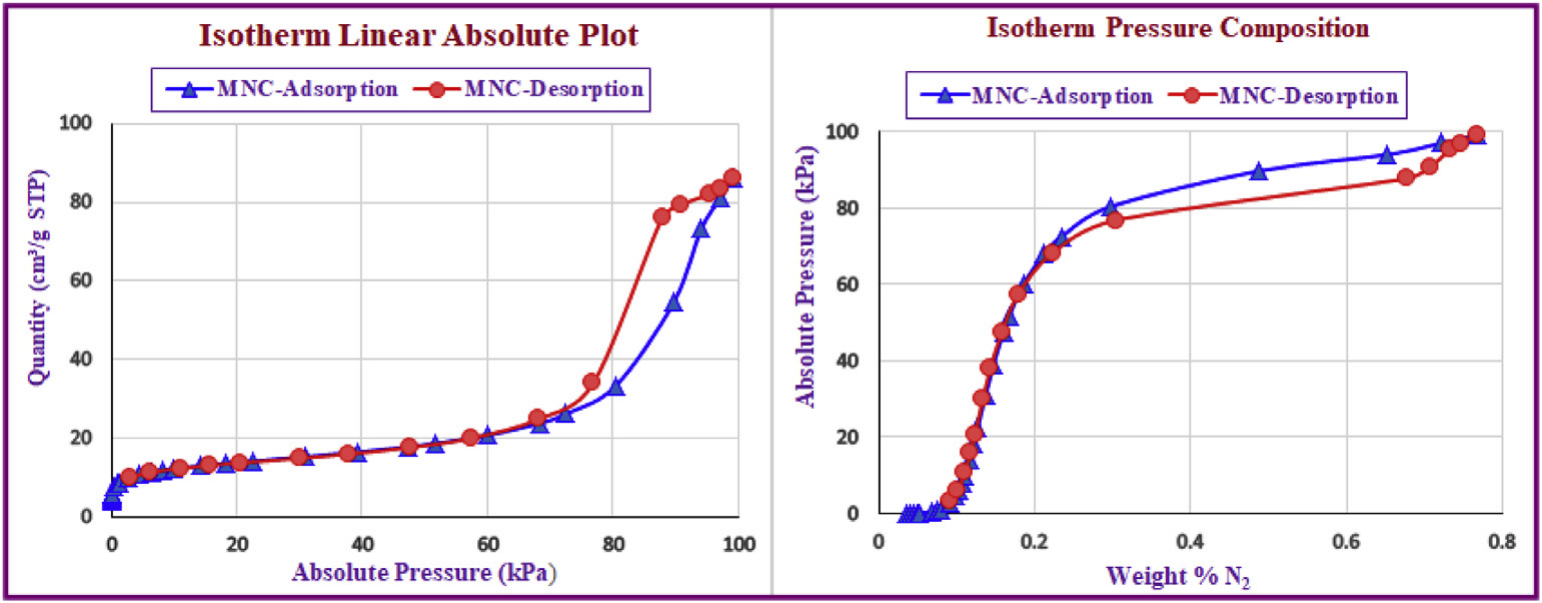
(a) Isotherm linear absolute plot (b) isotherm pressure composition.

**Fig. 5:**
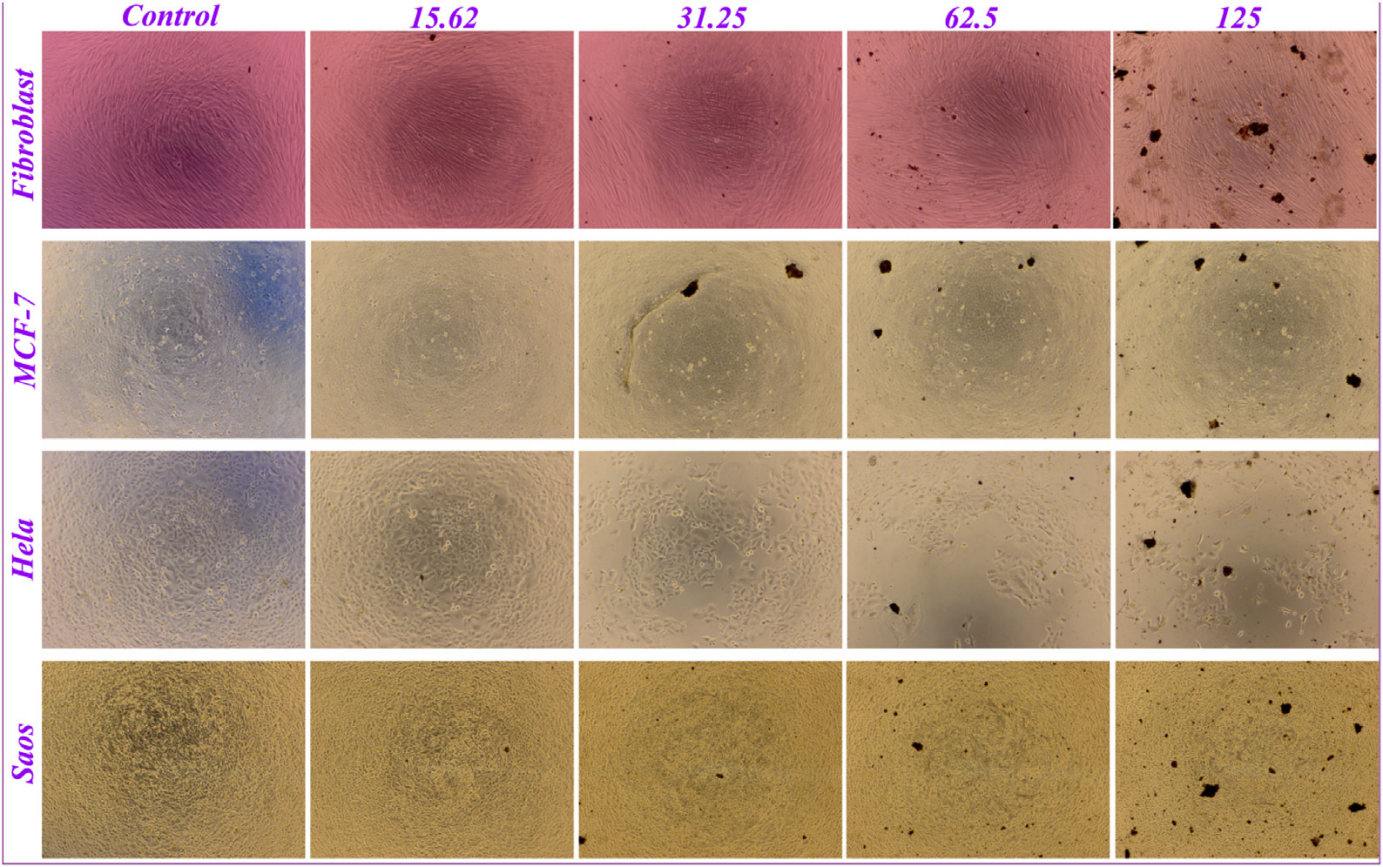
Morphology of the mentioned cell lines after incorporating the prepared nanobiocomposite samples in concentrations of 15.62, 31.5, 62.5 and 125 μg/mL.

IR spectra were recorded on a Perkin-Elmer FT-IR-1710 spectrophotometer with the samples in KBr pellets. Fig. 1 displays the FT-IR spectra of the prepared compounds and the characteristic peaks data were introduced as Table 1. Vibrating Sample Magnetometer (VSM) measurements were performed by using a vibrating sample magnetometer (LDJ Electronics Inc., Model 9600) and the data was inserted as Table 2 and the resulted pattern was displayed in Fig. 2. The X-ray powder diffraction (XRD) of the catalyst was carried out on a Philips PW 1830 X-ray diffractometer with CuKα source (λ = 1.5418 Å) in a range of Bragg's angles (5–80°) at room temperature and demonstrated in Fig. 3. The crystal planes of Fe_3_O_4_ which confirm the existence of magnetic nanoparticles in the composite were assigned in Table 3 Brunauer–Emmett–Teller (BET) analysis were performed using automatic sorption analyzer ASAP 2020, Micromeritics, USA. The Isotherm Linear Absolute Plot and isotherm Pressure Composition dataset were inserted in Table 4, Table 5 respectively and the adsorption and desorption graphs were displayed in Fig. 4. The morphology of cancer cells after treatment with different concentrations of nanocomposite were displayed in Fig. 5.

**Table 1:** FT-IR analysis data of a) Azidated chitosan, b) Chitosan-silane composite, c) Chitosan-silane clicked @ Fe_3_O_4_.

FT-IR Analysis	Wavenumber (cm^−1^)
Azidated chitosan *(a)*	910, 1090, 1160, 1270, 1395, 1540, 1625, 2100, 2895, 3430 (br)
Chitosan-silane composite *(b)*	1070, 1150, 1420, 1650, 2945, 3265, 3450
Chitosan-silane clicked @ Fe_3_O_4_*(C)*	570, 1018, 1107, 1400, 1620, 2925, 3400

**Table 2:** The detailed magnetization data versus applied field of MNC.

(Oe)	emu/g	(Oe)	emu/g	(Oe)	emu/g	(Oe)	emu/g	(Oe)	emu/g
1.90	−0.02	5501.11	24.62	321.11	13.21	−828.88	−18.44	−2469.45	−22.66
19.409	1.34	6001.11	24.78	236.11	11.40	−1057.49	−19.57	−1935.21	−21.89
33.72	2.44	6501.11	24.93	191.83	10.15	−1285.17	−20.37	−1545.17	−21.11
47.11	3.44	7001.11	25.07	157.97	9.11	−1545.17	−21.07	−1287.70	−20.42
62.11	4.46	7501.11	25.18	131.71	8.20	−1951.52	−21.87	−1051.93	−19.60
78.38	5.50	8001.11	25.29	111.58	7.39	−2469.45	−22.64	−828.88	−18.47
91.90	6.29	8501.11	25.40	91.11	6.49	−2998.88	−23.23	−743.88	−17.92
111.96	7.26	8001.11	25.29	78.04	5.74	−3498.88	−23.63	−658.88	−17.29
133.18	8.12	7501.11	25.18	61.75	4.71	−3998.88	−23.94	−573.88	−16.53
158.35	9.00	7001.11	25.06	45.95	3.68	−4498.88	−24.20	−488.88	−15.63
192.15	10.07	6501.11	24.92	32.08	2.70	−4998.88	−24.42	−403.88	−14.50
236.11	11.32	6001.11	24.81	17.14	1.56	−5498.88	−24.62	−318.88	−13.08
321.11	13.13	5501.11	24.63	0.50	0.20	−5998.88	−24.79	−239.66	−11.38
406.11	14.54	5001.11	24.44	−11.57	−0.72	−6498.88	−24.94	−175.97	−9.58
491.11	15.66	4501.11	24.22	−27.47	−1.96	−6998.88	−25.06	−140.75	−8.38
576.11	16.54	4001.11	23.96	−42.01	−3.01	−7498.88	−25.19	−113.56	−7.28
661.11	17.31	3501.11	23.65	−57.33	−4.00	−7998.88	−25.30	−91.45	−6.23
746.11	17.92	3001.11	23.26	−73.00	−4.95	−8498.88	−25.40	−72.20	−5.17
831.11	18.47	2501.11	22.75	−91.45	−6.01	−7998.88	−25.29	−57.41	−4.27
916.11	18.94	2001.11	22.07	−113.56	−7.04	−7498.88	−25.19	−40.59	−3.19
1178.04	20.00	1521.03	21.08	−141.48	−8.19	−6998.88	−25.06	−26.32	−2.21
1533.99	21.08	1173.47	20.03	−176.25	−9.47	−6498.88	−24.93	−11.81	−1.08
2001.11	22.04	916.11	18.97	−238.94	−11.30	−5998.88	−24.78	1.90	−0.02
2501.11	22.72	831.11	18.50	−318.88	−13.02	−5498.88	−24.63		
3001.11	23.23	746.11	17.97	−403.88	−14.41	−4998.88	−24.43		
3501.11	23.62	661.11	17.35	−488.88	−15.62	−4498.88	−24.20		
4001.11	23.94	576.11	16.60	−573.88	−16.47	−3998.88	−23.94		
4501.11	24.20	491.11	15.71	−658.88	−17.23	−3498.88	−23.63		
5001.11	24.42	406.11	14.60	−743.88	−17.896	−2998.88	−23.25

**Table 3:** Data resulted from XRD pattern of MNC.

	Crystal planes (2-Theta)													
*Fe*_*3*_*O*_*4*_	2 2 0 (30.4°)		3 1 1 (35.7°)			4 0 0 (43.4°)		4 2 2 (53.6°)			5 1 1 (57°)		4 4 0 (63.0°)	
Chitosan/SiO_2_	Broad Peak (15–30°)													
Unassigned	12.2	18		21.3	24.8		25.4		28	76

**Table 4:** Quantity of MNC-adsorption and desorption versus absolute pressure.

No.	MNC-Adsorption		MNC-Desorption	
	Quantity (cm³/g STP)	Absolute Pressure (kPa)	Quantity (cm³/g STP)	Absolute Pressure (kPa)
1	0.0155	3.9491	3.1219	10.2207
2	0.0308	4.5614	6.2336	11.3728
3	0.0533	5.0750	11.0299	12.4181
4	0.0775	5.4451	15.8141	13.1678
5	0.1012	5.7218	20.6030	13.8167
6	0.1165	5.8714	30.1617	15.0107
7	0.5385	7.7433	37.9474	16.0667
8	0.9008	8.4642	47.5964	17.7131
9	1.1723	8.8480	57.5541	20.1034
10	2.8142	10.1713	68.1428	24.9174
11	4.5636	10.9367	76.8254	34.0477
12	6.3321	11.4933	87.8111	75.9624
13	8.1536	11.9367	90.7318	79.2349
14	9.9705	12.3060	95.2962	82.0726
15	14.1124	13.0277	97.0361	83.6500
16	18.3035	13.6566	99.0819	86.1214
17	22.4814	14.2307		
18	30.8083	15.3401		
19	39.1600	16.5298		
20	47.4831	17.9148		
21	51.6722	18.7439		
22	59.9826	20.7978		
23	68.2312	23.8461		
24	72.5367	26.2876		
25	80.4362	33.3906		
26	89.6708	54.5202		
27	94.0723	73.1871		
28	97.0785	80.9530		
29	99.0819	86.1214

**Table 5:** Quantity of absolute pressure versus weight %N_2_.

No.	MNC-Adsorption		MNC-Desorption	
	Weight % N_2_	Absolute Pressure (kPa)	Weight % N_2_	Absolute Pressure (kPa)
1	0.0352	0.0155	0.0912	3.1219
2	0.0407	0.0308	0.1014	6.2336
3	0.0452	0.0533	0.1108	11.0299
4	0.0485	0.0775	0.1174	15.8141
5	0.0510	0.1012	0.1232	20.6030
6	0.0523	0.1165	0.1339	30.1617
7	0.0690	0.5385	0.1433	37.9474
8	0.0755	0.9008	0.1580	47.5964
9	0.0789	1.1723	0.1793	57.5541
10	0.0907	2.8142	0.2223	68.1428
11	0.0975	4.5636	0.3038	76.8254
12	0.1025	6.3321	0.6778	87.8111
13	0.1065	8.1536	0.7070	90.7318
14	0.1098	9.9705	0.7323	95.2962
15	0.1162	14.1124	0.7464	97.0362
16	0.1218	18.3035	0.7684	99.0819
17	0.1269	22.4814		
18	0.1368	30.8083		
19	0.1474	39.1600		
20	0.1598	47.4831		
21	0.1672	51.6722		
22	0.1855	59.9826		
23	0.2127	68.2312		
24	0.2345	72.5367		
25	0.2979	80.4362		
26	0.4864	89.6708		
27	0.6530	94.0723		
28	0.7223	97.0785		
29	0.7684	99.0819

## 2 Experimental design, materials and methods

The magnetic nanocomposite was prepared using chitosan. To prepare functionalized chitosan, the chitosan was azidated using chloroacetyl chloride and sodium azide. Then click reaction which has been incorporated in our recent studies [2,3] and also employed in some biological researches [4,5] was performed between functionalized chitosan and trimethoxy(3-(prop-2-yn-1-ylthio)propyl)silane. Then magnetization was done using ferric and ferrous chloride solution. The characteristic peaks for azidated at around 2100 cm^−1^, C–H bond of triazole rings and Si–O–Si bonds at 3265 cm^−1^ and 1150 cm^−1^ respectively. The resulted FT-IR spectra and the corresponding data of the synthesized products were presented in Fig. 1 and Table 1. Also, the detailed FT-IR data including the transmittances at each wavenumbers for the compounds a, b, and c were provided as a supplementary file.

Magnetization experiments of the prepared magnetic nanocomposite (MNC) were obtained using VSM technique at room temperature. As can be seen in Fig. 2, this product with saturation magnetization value (M_s_) of 25.4 (emu/g) has super paramagnetic characteristics. Moreover; the corresponding data were presented in Table 2.

The XRD pattern of the synthesized chitosan nanocomposite (Fig. 3) demonstrated the crosslinking reaction between Si groups and chitosan with the broad peak at 15–30°. Moreover the existence of magnetic nanoparticles in the structure was confirmed by determining the crystal planes of Fe_3_O_4_ nanoparticles (Table 3).

To attain adsorption data of the synthesized nanocomposite for further experiments, the samples were outgassed at 60 °C and then experiments according to the Brunauer–Emmett–Teller (BET) theory were performed. The isotherm plots were used to calculate the specific surface area and the average pore diameter of the chitosan/magnetic nanocomposite and the difference between adsorption and desorption steps.

For evaluating the cell cytotoxicity of the prepared sample (MNC) according to the literature [6], some known cell lines were considered including Fibroblast, MCF7, Hela, and Saos. The resulted data were surveyed in the main article and the morphology of the cell lines with treatment of different concentrations of the samples were imaged by microscopic technique and presented here in Fig. 5.
